# Rational design of FVIII sialylated peptides to target Siglec-3 and Siglec-9 and improve peptide formulations for reverse vaccines

**DOI:** 10.3389/fbioe.2025.1558627

**Published:** 2025-04-10

**Authors:** Eleonora Nardini, Brigitte-Carole Keumatio Doungstop, Tania Gerpe-Amor, Aram M. de Haas, Mike de Kok, Evert Peterse, Hakan Kalay, Rui-Jún E. Li, Fabrizio Chiodo, Alba Silipo, Jan Voorberg, Yvette van Kooyk

**Affiliations:** ^1^ Amsterdam UMC location Vrije Universiteit Amsterdam, Molecular Cell Biology and Immunology, Amsterdam, Netherlands; ^2^ Amsterdam institute for Immunology and Infectious diseases, Immunology, Amsterdam, Netherlands; ^3^ Department of Chemical Sciences and Task Force for Microbiome Studies, University of Naples Federico II, Naples, Italy; ^4^ DC4U Technologies, Abcoude, Netherlands; ^5^ Institute of Biomolecular Chemistry, National Research Council (CNR), Naples, Italy; ^6^ Department of Molecular Hematology, Sanquin Research, Amsterdam, Netherlands

**Keywords:** hemophilia a, FVIII, inhibitors, sialic acid, siglec-9, peptide immunotherapy, tolerance, dendritic cells

## Abstract

Reverse vaccine formulations have shown their potential for the treatment of allergies and other autoimmune diseases by the design of antigens that modify dendritic cell function towards tolerogenic responses. We here demonstrate that modification of an immunodominant peptide from factor VIII (FVIII) with a tolerizing molecule, sialic acid, improves existing peptide formulations towards the induction of tolerogenic cytokine secretion by DCs. Sialic acids are the end-standing moiety of mammalian N- and O- glycans, which are naturally recognized as self-associated molecular pattern. In this paper we show that sialic acid modified FVIII peptides target Siglec-3 and -9 on DCs and increase IL-10 secretion. Our work proposes a method to select, synthetize and test sialylated immunodominant peptides with the aim of ameliorating the efficacy of peptide immunotherapy. Based on our results, we propose that the sialylated FVIII peptide designed in this study may be useful for re-establishing tolerance to FVIII in hemophilia A patients who developed neutralizing antibodies following treatment.

## Graphical Abstract

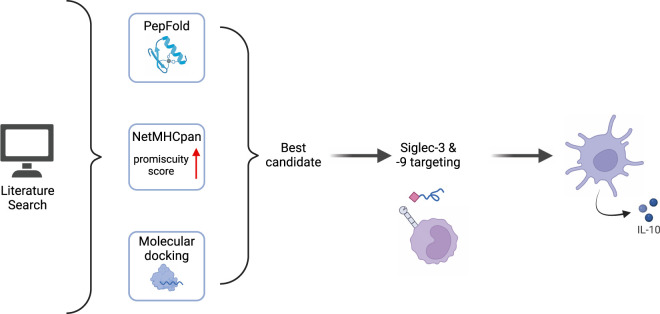

## 1 Introduction

Peptides are used in reverse vaccination as an alternative to whole-antigen formulations to achieve tolerance in allergies and autoimmune diseases, as demonstrated by the ongoing clinical trials in type 1 diabetes (Van Rampelbergh et al., 2023), Graves’ (Pearce et al., 2019) and MS (Chataway et al., 2018). The use of immunodominant sequences allows for the reduction of costs and risks associated with infusing the full antigens, while still maintaining control over the amounts injected. Recently, peptide immunotherapy has been tested in preclinical models of hemophilia A (HA) (Pletinckx et al., 2022), which is an X-linked genetic disorder caused by mutations of coagulation factor VIII (FVIII) (Mannucci and Franchini, 2014). Although the quality of life of these patients significantly improved in the last decades, up to 30% of them still develop neutralizing antibodies to FVIII, called inhibitors, hampering the success of the treatment. Currently, the only available option to eradicate the inhibitors is Immune Tolerance Induction (ITI), which consists of repeated infusions of FVIII at high doses for a prolonged period of time. ITI poses a high burden on the patients and the healthcare system, and success rate varies between 60% and 90% (Oomen et al., 2023). Better alternatives to promote tolerance to FVIII are therefore needed, aiming at 100% eradication of the inhibitors while using limited amount of antigen and fewer injections. A recently published approach which employs FVIII peptides has proved promising in an experimental model of HA (Pletinckx et al., 2022). The strategy from Pletinckx *et al.* is based on the use of antigen processing-independent T cell epitopes (apitopes) (Pletinckx et al., 2022). Strict chemical requirements allow apitopes to directly bind MHC-II on steady state APCs in lymphoid organs. In the absence of concomitant danger signals, these immature APCs presents antigens to T cells in a tolerogenic way, skewing them to type 1 regulatory T cells (Tr1s). Tr1s express inhibitory receptors (CTLA4, TIM3, LAG3, TIGIT) and mostly secrete IL-10, creating a microenvironment that suppresses the expression of co-stimulatory molecules on neighboring APCs and promotes the generation of IL-10-secreting B_regs_ and myeloid-derived suppressor cells (Pletinckx et al., 2022). However, complete eradication of the inhibitors was not achieved with apitopes in the preclinical models and the protocol required frequent injections, which will likely affect patients’ compliance. To improve the efficacy of such peptide formulations, we propose to modify FVIII peptides by chemically conjugating them to a tolerogenic signal, sialic acid (sialylation). Sialic acids are monosaccharides capping the majority of mammalian glycans (Varki et al., 1999). Sialylated glycans are the ligands for immunosuppressive receptors called Sialic-acid-binding immunoglobulin-like lectins (Siglecs), broadly expressed by immune cells (Crocker et al., 2007). The majority of Siglecs signals through an Immunoreceptor tyrosine-based inhibitory motif (ITIM) (Angata et al., 2022). Upon binding to sialylated ligands, phosphorylation of the ITIM by adaptor proteins, mainly SHP1 and 2, initiates a cascade that culminates in inhibition of NFkB translocation in the nucleus, resulting in immune suppression (Angata et al., 2022). The family of Siglecs comprise fifteen lectins, which differ in their preference of binding to specific sialoglycans and their pattern of expression in immune subsets (Angata et al., 2022). Targeting Siglec-9 with sialylated dendrimers induced a tolerogenic profile in monocyte derived dendritic cells (moDCs) *in vitro* (Lübbers et al., 2021). Moreover, in the context of allergies, sialylation of the house dust mite allergen reduced the detrimental secretion of Th2 cytokines from PBMCs (Keumatio Doungtsop et al., 2024) and treatment with sialylated peptides ameliorated the symptoms of grass pollen allergy *in vivo* (Hesse et al., 2019). In this study, we present an improvement of sialylated peptide formulations by rational design of the peptide sequence, in order to target the largest portion of population, and of the sialylated glycan to improve targeting to Siglec-9. We demonstrate that our implemented peptide formulation binds both to Siglec-3 and -9. We further show that stimulation of monocyte-derived DCs (moDCs) with sialylated peptides induces IL-10 secretion in a Siglec-3 and Siglec-9 dependent manner. IL-10 was shown to be a key signal in the response to FVIII to restore and maintain tolerance (Karim et al., 2020), suggesting that our FVIII peptide formulation might prove useful in the treatment of HA patients with inhibitors.

## 2 Methods

### 2.1 Peptide synthesis and conjugation to the sialylated glycan and to TAMRA

FVIII derived peptide (KHNIFNPPIIARYIRLHPTHYSIRST; residues 2,155–2,180) was synthesized by solid-phase using Fmoc chemistry on a CEM Liberty Blue peptide synthesizer and conjugated to Neu5Acα2-3Galβ1-4GlcNAcβ1-3Galβ1-4Glc (LSTd) and 5(6)-Carboxytetramethylrhodamine (TAMRA, Sigma). The detailed method is described in the supplementary materials.

### 2.2 *In silico* analyses

Predictions of the binding affinities to various HLA-DRB alleles were computed using the publicly available software NetPanMHCII4.0 (Reynisson et al., 2020), while PEP-FOLD_3_ was used to predict the secondary structures (Lamiable et al., 2016).

#### 2.2.1 Ligand preparation for computational analysis

The 3D coordinates for Sia were generated using the GLYCAM database. Subsequently, the ligand geometries underwent optimization through molecular dynamics (MD) simulations. Siglec-9 model was built using AlphaFold (Jumper et al., 2021). An AlphaFold homology model was used to run MD simulations. MD simulations were carried out with AMBER18 package implemented with the ff14SB for proteins. As for natural glycan moieties, the GLYCAM06j-1 forcefield was used.

#### 2.2.2 Docking calculations

Docking calculations of the glycan were performed using AutoDock 4.2 (Morris et al., 2009). The docking protocol was validated by using the AlphaFold modelled structure from Siglec-9. A total of 200 runs using Lamarckian Genetic algorithm was performed, with a population size of 100. After docking, the 100 solutions were clustered in groups with root-mean-square deviation less than 1.0 Å. The clusters were ranked to the lowest energy representative of each one.

#### 2.2.3 MD simulations

MD simulations were conducted using AMBER 18 with explicit water and specific forcefields (Glycam06j-1 for glycans, FF14SB for proteins, and Gaff for organic moieties) (Case et al., 2018). Proteins were prepared by adding missing hydrogens, setting ionizable group states, and capping termini. Input files were created using the AMBER tleap module, energy minimizations were done with Sander, and MD simulations were performed with PMEMD. Systems were placed in a TIP3P water box, neutralized with counterions, and refined using energy minimization. Simulations included heating (0–300 K), equilibration, and a 100 ns production run with 1 fs timesteps. Trajectories were analyzed for stability and structural properties using Cpptraj, and visualized with PyMOL.

### 2.3 Validation of sialylation and binding to Siglecs

The presence and functionality of the sialylated glycan was confirmed by ELISA with α2,3-specific Lectenz (Lectenz Bio), Maackia Amurensis Lectin I (MAL-I) (Vector Laboratories), and Sambucus Nigra Lectin (SNA, EBL) (Vector Laboratories), while binding to Siglecs was similarly assessed by ELISA with Siglec-Fc chimeras. The detailed methods are described in the supplementary materials.

### 2.4 Binding/uptake experiments of TAMRA-(sia)FVIII_2155-2180_

PBMCs were isolated from the buffy coats of healthy donors (Sanquin, reference: S03.0023-XT) by Ficoll density gradient and monocytes were obtained by Percoll density gradient. These were cultured for 5 days to generate monocyte-derived dendritic cells (moDCs), as previously described (Bax et al., 2011). At day 5 of differentiation, 0.1 × 10^6^ moDCs per time point incubated on ice for 30 min with 10 µM of TAMRA-FVIII_2155-2180_ or TAMRA-siaFVIII_2155-2180_ diluted in blocking buffer (HBSS (Invitrogen) + 0.5% BSA). Next, 0.1 × 10^6^ moDC were placed on ice as time point zero, while the rest of the sample was incubated at 37°C for the number of minutes indicated in Figure 3E. After the last incubation step, cells were washed twice with blocking buffer, stained with the fixable-viability-dye efluor780 (Invitrogen), fixed in 1% PFA and measured at the LSRFortessaX-20 (BD Bioscience). The data were analyzed with FlowJo (v10) by gating on the alive cells and extracting the MFI of TAMRA. Every time point of each peptide (FVIII_2155-2180_ or siaFVIII_2155-2180_) was normalized over time point 0.

### 2.5 Phosphorylation analysis

The phosphorylation of Siglec-9 induced by sialylated peptides in monocytes was assessed with the Human Phospho-immunoreceptor array kit (R&D systems) according with manufacturer’s instructions. For the generation of the cell lysates, 1 × 10^5^ CD14^+^ isolated monocytes were first starved in FSC-free RPMI-1640 medium for 4 h at 4°C and then stimulated for approximately 15 min with 10 μM FVIII_2155-2180_ or siaFVIII_2155-2180_ at 37°C, previously coated overnight at 4°C in a 24-well plate. The cell lysates were then obtained with the kit’s buffers, their concentration was determined with the micro-BCA protein assay kit (Thermo Scientific) and 50 μg were used for the array.

### 2.6 Generation of the knock-outs

One million monocytes were obtained from PBMCs of healthy donors by positive selection using CD14^+^ MACS beads (Miltenyi) and then nucleofected with 2.5 μL of a 20 μM solution of precomplexed RNPs, as previously described (Hiatt et al., 2021). Briefly, stock solutions of 20 μM RNPs were generated by incubating equal volumes of 40 μM Cas9 nuclease protein NLS (Horizon CAS12206) and 80 μM gRNAs at 37°C for 15 min. These were then immediately used or stored at −80°C. GuideRNAs targeting Siglec-3 CGG​TGC​TCA​TAA​TCA​CCC​CA and Siglec-9 CCC​TCT​CCC​TCC​CCC​AGA​GC were synthesized by Genscript using their CRISPR gRNA Synthesis Service. Mock monocytes were obtained by precomplexing tracrRNA (Dharmacon, U-002005–0,050) with Cas9 and double KO were generated by mixing equimolar ratios of single gRNAs to a final concentration of 80 μM. After nucleofection, the monocytes were cultured for 5 days to generate moDCs as described in paragraph 2.4.

### 2.7 Cytokines secretion from moDCs and surface marker expression

At day 5 of differentiation, 0.5 × 10^5^ wild type, mock, Siglec-9, Siglec-3/-9 double knock-out moDCs were stimulated with 10 µM of FVIII_2155-2180_ or siaFVIII_2155-2180_ and matured overnight with 10 ng/mL LPS (LPS-EB Ultrapure from *E. coli* O111:B4, Invivogen). Samples without LPS were taken along as controls. The supernatants were collected and stored at −20°C to be processed with LEGENDplex™ Human Inflammation Panel 1 (13-plex) (BioLegend, 740,809), which was then performed according with the manufacturer’s instructions. Cytokine concentrations were determined using BioLegend’s LEGENDplex™ data analysis software, while the cells were processed for flow cytometry analysis. Briefly, the cells were incubated on ice for 15 min with fixable viability dye eFluor450 (Thermo Fisher) and Fc-block (BD Biosciences). Then, moDCs were stained for 30 min on ice with the antibody mix (Supplementary Table S2) diluted in PBS +0.1% BSA (Roche) + 0.02% sodium azide. Finally, samples were fixed in 1% PFA, measured at the LSRFortessa X-20 and analyzed with FlowJo (v10).

### 2.8 Statistics

All tests were performed using Graphpad PRISM version 10 or the R package ggstats (Larmarange, 2024). P-values ≤0.05 were considered significant.

## 3 Results

### 3.1 Selection of the peptide sequence

**FIGURE 1:**
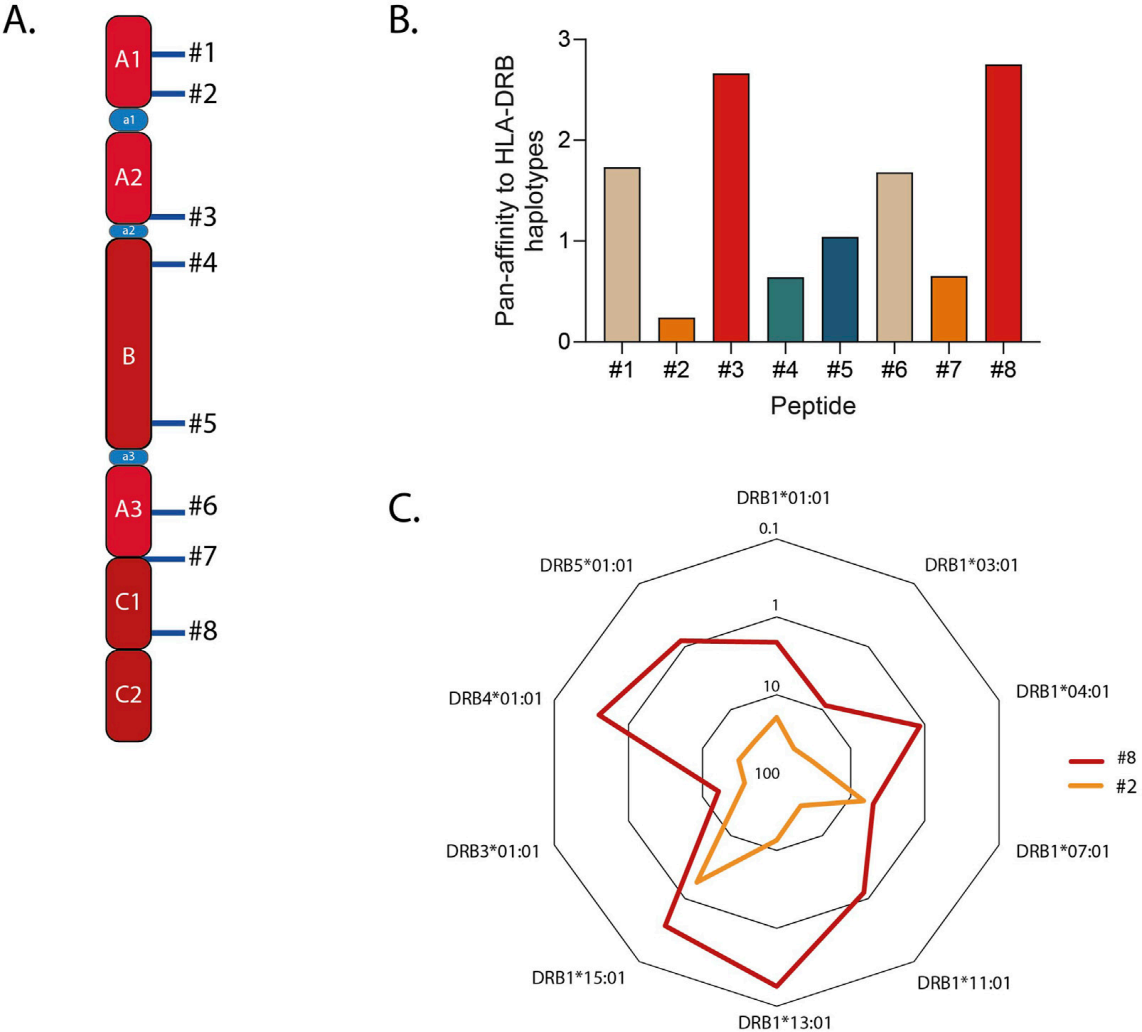
Selection of the FVIIII sequence to be conjugated to the Siglec-9 ligand (Sia). **(A)** Schematic showing the immunodominant sequences identified in Steinitz et al. (2012). **(B)** Promiscuity of binding to the most common HLA haplotypes in the Caucasian population, plotted as the inverse of the sum of ranks predicted *in silico*. **(C)** The closed hexagonals shown in the radar charts represent the percentile rank (ranging from 0.1% to 100%) to each HLA-DRB allele for peptides #2 and #8, representative of the most and the least promiscuous peptides respectively.

To choose the peptide sequence, we shortlisted FVIII immunodominant T cell epitopes identified by Steinitz and colleagues (Steinitz et al., 2012) (Figure 1A; Supplementary Table S3). For each core peptide, *i.e.*, the 21-aminoacid sequence fitting into the MHC-II groove, the prediction of binding to the most common HLA-DRB alleles in the Caucasian population (Alexander et al., 1994; Panina-Bordignon et al., 1989) was computed using NetMHCIIpan4.0 (Reynisson et al., 2020). The percentile rank score was used to summarize the promiscuity of binding of every sequence. This value represents the predicted affinity of the input sequence compared to a set of 100,000 random natural peptides, it is thus not inherently biased. We then calculated the sum of ranks to each HLA-DRB haplotype and plotted it as the inverse figure (Figure 1B). Core sequence #8 scored as the most promiscuous, being classified among the top 1% binders for four alleles and within the top 17% for the others (Figure 1C). As expected, all the sequences are predicted to be strong binders for DRB1*15:01, since they were identified by Steinitz *et al.* in a mouse model humanized with this haplotype. This confirms the reliability of the percentile rank score as a proxy measure for promiscuousness. Based on these results, we selected core peptide #8 and synthesized a 25-mer peptide spanning residues 2,155–2,180 of FVIII, which included core sequence #8. This longer peptide was designed to require antigen processing by APCs prior to MHC-II presentation, thereby preventing direct binding of the core peptide to empty MHC-II molecules. In the following sections, this peptide is referred to as “FVIII_2155-2180_ “. We next interrogated the IEDB software to compute population coverage worldwide for FVIII_2155-2180_, which resulted to be higher than 75% (Bui et al., 2006). Overall, these findings underscore the rationale for selecting core peptide #8 and designing the extended FVIII_2155-2180_ peptide, which demonstrated broad predicted HLA-DRB binding promiscuity and global population coverage.

### 3.2 Selection of Siglec-9 ligand and conjugation to FVIII_2155-2180_

**FIGURE 2:**
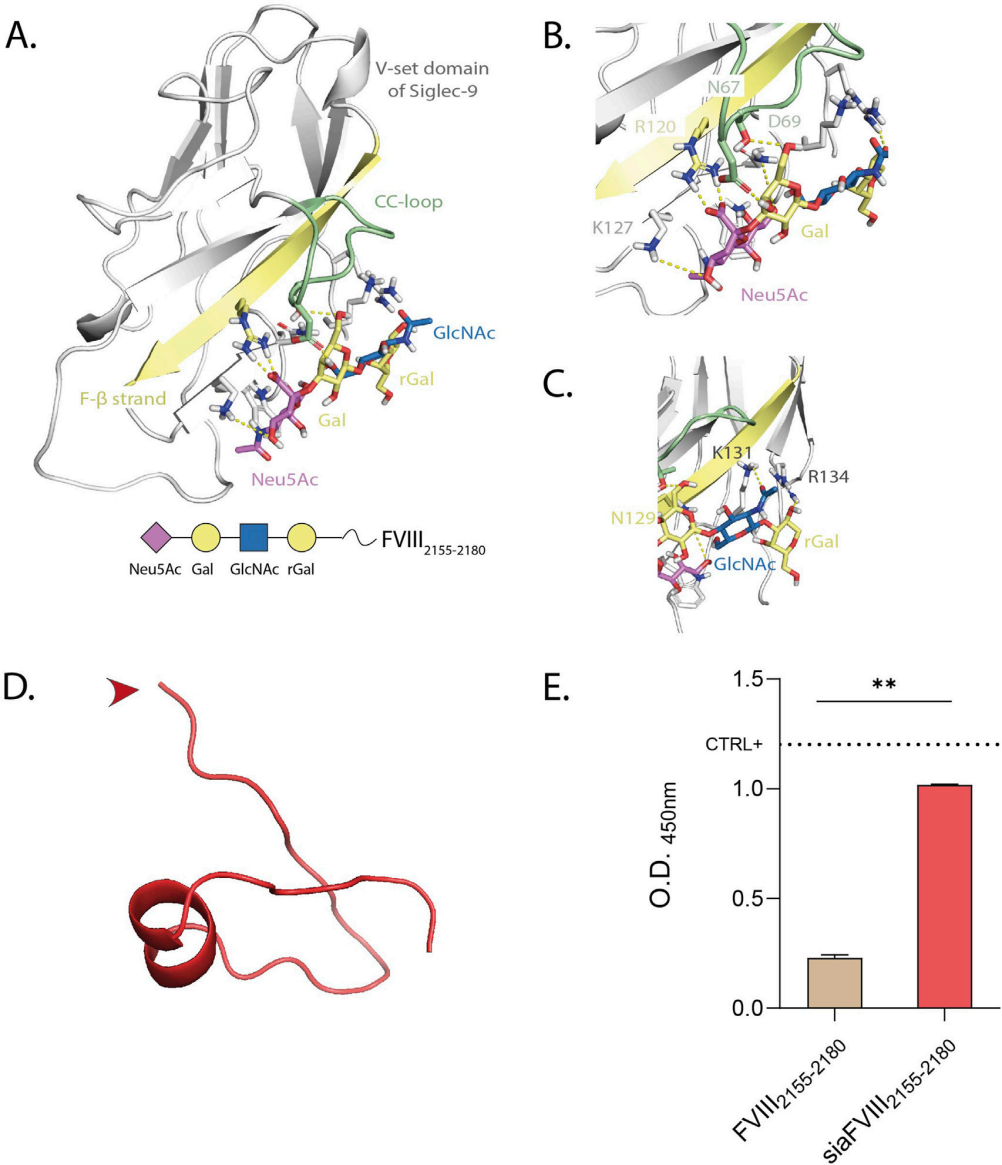
Selection of Siglec-9 ligand (Sia) and conjugation to FVIII. **(A)** Different views highlighting H-bonds between the 3D model of Siglec-9 and the Sia, monitored by MD simulation. The strands involved in the recognition are colored by type: F-β strand in yellow and CC-loop in green. The ligand is represented following SNFG color-code. B, **(C)** Close up views of the interactions between Siglec-9 and the first **(B)** or second half **(C)** of the sialoglycan. **(D)** PEP-FOLD3 prediction of the secondary structure of FVIII_2155-2180_ in physiological conditions. The red arrow indicates the site of conjugation to Sia. **(E)** α2.3PanLectENZ binding to siaFVIII_2155-2180_. The dotted line marks the mean O.D. value of α2.3 PAA used as a positive control. Representative of 3 independent experiments performed in duplicate. Statistics: unpaired t-test.

We then conjugated FVIII_2155-2180_ to sialic acid (Neu5Ac), to target it to the immune inhibitory receptor on DCs, Siglec-9 (Zhang et al., 2000). Siglec-9 belongs to a family of 15 lectins, the majority of which carries an intracellular inhibitory motif to suppress the immune response upon binding to sialic acid. Siglecs show preferential binding to different sialylated glycans. It is known that Siglec-9 preferentially binds N-glycans with terminal α2.3-Neu5Ac (Briard et al., 2018; Narimatsu et al., 2019; Blixt et al., 2004; Rodrigues et al., 2020), so we used the tetrasaccharide Neu5Acα2-3Galβ1-4GlcNAcβ1-3Gal (Sia) to chemically modify FVIII_2155-2180_ (depicted in Figure 2A and addressed to as siaFVIII_2155-2180_ in the following sections). To study the interactions of Sia with Siglec-9, docking to the V-set domain of Siglec-9 was performed. The interactions were mapped to propose a molecular description of the complex (Figures 2A–C). The 3D models showed a stable salt-bridge interaction between the carboxylate group of Neu5Ac and the guanidinium group of R120 along the simulation (Figure 2B). Neu5Ac established polar interactions involving the O9 of its glycerol chain, through the formation of H-bonds with the lateral chain of N129. An additional contact from O7 of the glycerol chain with K127 was also observed (Figure 2B). The results from the MD simulation further showed that D69 from the *CC*-loop and K131 and K127 from the F-β strand were highly implicated on the accommodation of Gal, stabilizing the ligand into the binding pocket (Figures 2B,C). Specifically, the position O6 of Gal established a hydrogen bond with the lateral chain of N67 and its O4 with D69. The GlcNAc and the reducing Gal established transient interactions within K127 and R134, respectively. This suggests that this is the particular position where the following peptide FVIII_2155-2180_ is coupled (Figure 2A). We next modelled the secondary structure of FVIII_2155-2180_ with PepFold3, of which one representative conformation is shown in Figure 2D. Amino acids from P2143 to Y2148 formed an alpha helix. We reasoned that it would be an advantage to guarantee flexibility and availability of binding to Siglec-9 to conjugate Sia to the N-terminus, indicated by the red arrow, given that it is not involved in the helix. We subsequently tested for the functionality of Sia after chemical conjugation to the peptide by ELISA. We observed that siaFVIII_2155-2180_ but not FVIII_2155-2180_ bound to α2.3PanLectENZ (Figure 2E) and to MAL-1 but not to SNA (Supplementary Figure S2A-B), known to only recognize α2,6 sialic acids and used as a negative control. In conclusion, sialylation at the N-terminus of FVIII_2155-2180_ with Sia demonstrated strong *in silico* engagement with the V-set domain of Siglec-9 and enhanced the binding of α2.3-specific lectins.

### 3.3 siaFVIII_2155-2180_ efficiently binds to Siglec-3 and Siglec-9 and it is bound/internalized by moDCs

**FIGURE 3:**
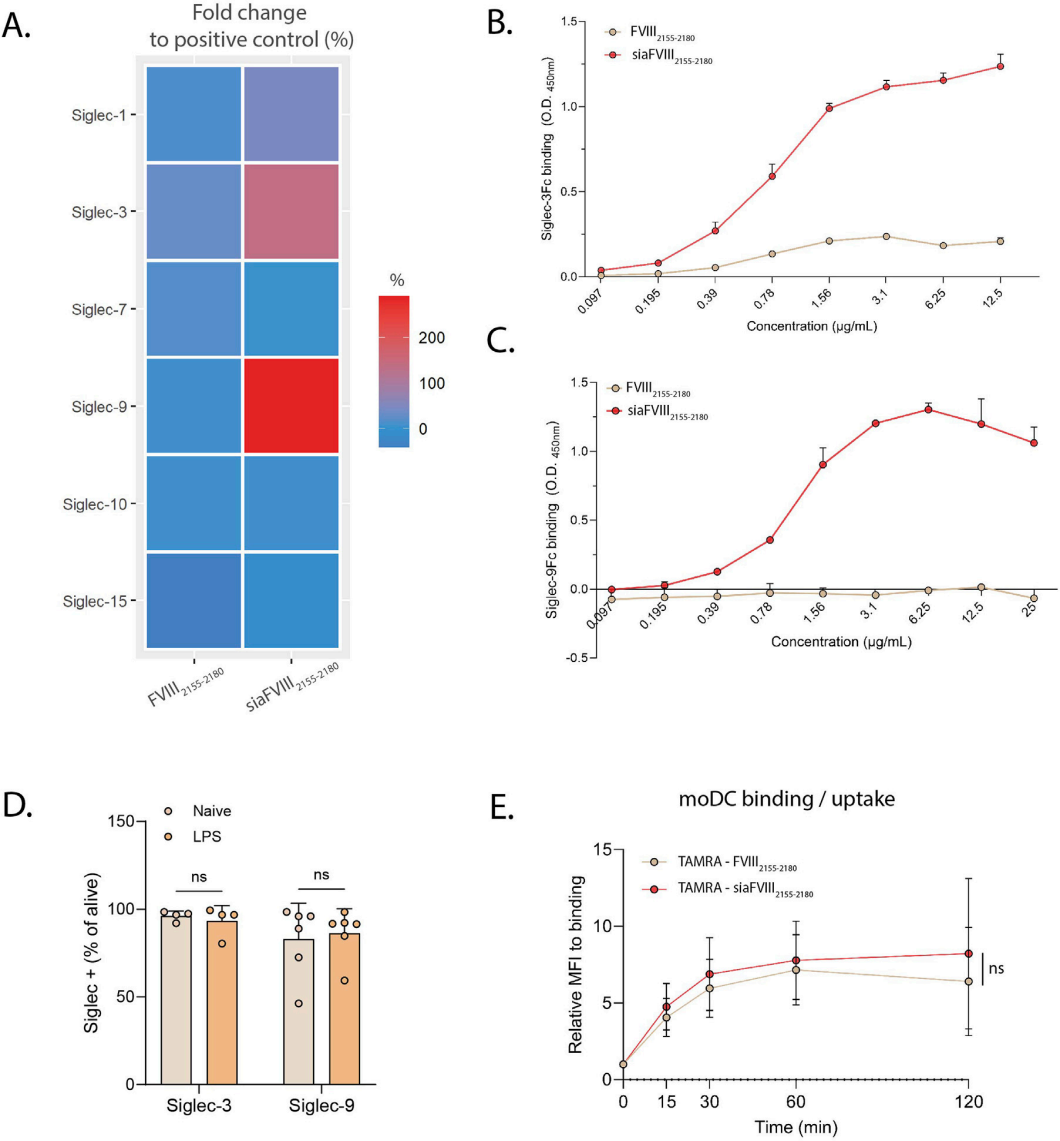
SiaFVIII_2155-2180_ efficiently binds to Siglec-3 and Siglec-9. **(A)** Heatmap showing binding of siaFVIII_2155-2180_ to various Siglec-Fc chimeras. The O.D. values are plotted as fold change to the positive control, set as 100% of binding. Data are presented as mean of three independent experiments. B, **(C)** Concentration dependent curves of binding to Siglec-3 **(C)** and Siglec-9 **(D)** of siaFVIII_2155-2180._ Data are shown as mean ± SD of two technical duplicates of three independent experiments.D. Siglec-3 and Siglec-9 expression in unstimulated moDCs (naïve) and moDCs stimulated overnight with 10 ng/mL LPS assessed by flow cytometry. The data represent the percentages of Siglec-positive cells, expressed as mean of 4 (Siglec-3) or 6 (Siglec-9) donors ± SD. Each dot represents one donor. Statistics: paired t-test, ns = not significant. **(E)** Binding/uptake of TAMRA-(sia)FVIII_2155-2180_ in moDCs measured by flow cytometry. Median fluoresce intensities (MFIs) are plotted as relative to time point 0 (binding), as mean of 8 donors ± SD. Statistics: paired t-test, ns = not significant.

Dendritic cells are the main immune subtype involved in antigen presentation to T cells and they express several Siglecs, Siglec-1, -3, -7, -9, -10 and −15 (Lübbers et al., 2018). We tested these Siglecs by ELISA for binding to siaFVIII_2155-2180_ observing that siaFVIII_2155-2180_ bound Siglec-9, and also engaged Siglec-3 (Figure 3A). A concentration dependent-binding was observed both for Siglec-3 and Siglec-9 to siaFVIII_2155-2180_ (Figures 3B,C), further confirming that both the lectins can recognize the sialylated glycan on the peptide backbone. To investigate the capacity of siaFVIII_2155-2180_ to be internalized by DCs, binding/uptake to monocyte-derived DCs (moDCs) was investigated by flow cytometry with (sia)FVIII_2155-2180_ fluorescently labelled with TAMRA. Siglec-3 and Siglec-9 expression by moDCs was confirmed by flow-cytometry in unstimulated (naïve) and LPS-matured moDCs (Figure 3D; Supplementary Figure S3A). Naïve cells were incubated for 30 min with TAMRA(sia)FVIII_2155-2180_ at 4°C and then at 37°C for different time points. Given that the variability between donors was great (Supplementary Figure S3C), data were normalized over time point 0, addressed to as “binding” (Figure 3E). From the resulting curves, it can be inferred that moDCs are equally able to bind/internalize FVIII_2155-2180_ and siaFVIII_2155-2180_.

### 3.4 siaFVIII_2155-2180_ activates Siglec-9 on monocytes and promotes IL-10 secretion from dendritic cells via Siglec-3 and Siglec-9

**FIGURE 4:**
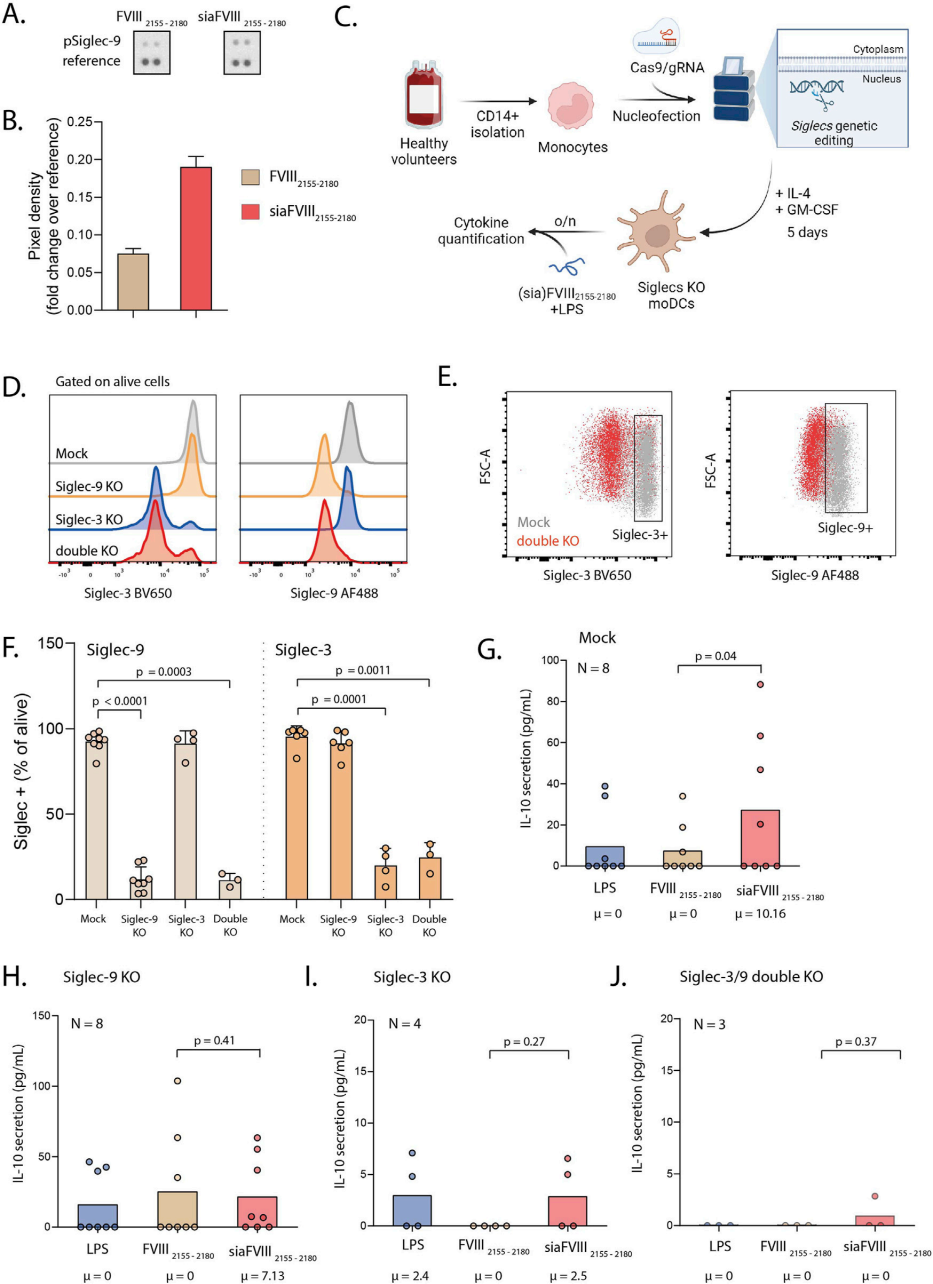
Stimulation of monocytes with siaFVIII_2155-2180_ activates Siglec-9 and promotes IL-10 secretion via Siglec-3 and Siglec-9. **(A)** Phospho-Immunoreceptor array of monocytes from one donor stimulated with siaFVIII_2155-2180_ or FVIII_2155-2180_. **(B)** Quantification of A, plotted as pixel density analyzed with Fiji. Data are presented as **(C)** Schematic of the experimental workflow; monocytes were nucleofected with Cas9 pre-complexed with gRNAs targeting Siglec-9 or a combination of Siglec-9 and Siglec-3. The cells were differentiated to moDCs for 5 days and then stimulated overnight with siaFVIII_2155-2180_ in the presence of LPS. Illustration was generated with biorender.com. **(D)** Histograms showing Siglec-3 (left) and Siglec-9 (right) expression across moDC mock, Siglec-9, Siglec-3 and double KO. **(E)** Gating strategy used to select Siglec-9 and Siglec-3 positive cells. **(F)** Percentages of Siglecs KO in the moDC cultures, analyzed by flow cytometry. **(G-J)** IL-10 secretion in mock, Siglec-9 single KO, Siglec-3 single KO and Siglec-3 and -9 double KO respectively, quantified with cytokine bead assay. Data are presented as box plots, the median is specified in text underneath. Statistics: Durbin-Conover pairwise test with Holm-Bonferroni correction for multiple comparisons. Each dot is the mean of two technical duplicates per donor, number of donors is specified in text in each graph. µ = median.

We then tested whether siaFVIII_2155-2180_ could activate Siglecs. We used tyrosine phosphorylation as marker for activation of Siglecs on monocytes stimulated with (sia)FVIII_2155-2180_ tested with an array of immunoreceptors blotted on a membrane and detected with anti-phophoTyr-HRP. Interestingly, siaFVIII_2155-2180_ selectively induced Siglec-9 phosphorylation (Figures 4A,B; Supplementary Figure S1A-B), indicating that Siglec-9 is the main receptor targeted by the sialylated peptide. To further explore the functional modulatory properties of the immune response induced by Siglec-targeting with siaFVIII_2155-2180_, we analysed maturation markers and cytokine secretion in LPS-matured moDCs. Primary monocytes knocked out of Siglec-9, Siglec-3, or a double KO of both Siglec-9 and Siglec-3 were generated by CrisprCas9 (Figures 4C–F), cultured for 5-day with IL-4 and GM-CSF to generate moDCs and then stimulated overnight with FVIII_2155-2180_ or siaFVIII_2155-2180_ in the presence of LPS. Binding of TAMRA(sia)FVIII_2155-2180_ to the mock and KO cells was confirmed by flow cytometry (Supplementary Figure S1C-D). No differences were detected in the expression of maturation (CD80, CD86) or tolerogenic (TIM3, PDL1) markers (Supp. Fig. E–H). A panel of essential cytokines was tested by bead array, of which IL-10 was the only cytokine showing significant differences between the groups (Supplementary Figure S1I). In mock moDCs, siaFVIII_2155-2180_ induced IL-10 secretion (Fig.G), while in the Siglec-KO cells this effect was not observed (Figures 4H–J), suggesting that functionally both Siglecs are needed to achieve immune modulation with siaFVIII_2155-2180_.

## 4 Discussion

The objective of the present study was to develop a novel FVIII-peptide formulation with improved targeting to two immune inhibitory receptors, Siglec-3 and Siglec-9 on DCs to be employed as reverse vaccines for hemophilia A patients with inhibitory FVIII antibodies.

*In silico* analyses of FVIII core peptides’ binding promiscuity to frequent Caucasian HLA-DRB alleles shortlisted a sequence predicted to cover 75% of the global population (Figures 1A–C). This estimate should be confirmed *in vitro* by employing an array of MHC-II molecules. However, independent studies that characterized the immunodominant peptidome of FVIII using mass spectrometry identified peptides within the FVIII_2155-2180_ region across various donors (van Haren et al., 2011; Diego et al., 2020). Moreover, T cells reactive with peptide I2163-T2,179, within FVIII_2155-2180_, were isolated from an HA patient (Jacquemin et al., 2003). Altogether, this evidence reinforces our results. Population coverage could be increased by synthetizing a pool of epitopes, given that chemical coupling of Sia is potentially feasible for every FVIII epitope, but using one sequence enhances scalability and standardization. Moreover, chemical synthesis is preferable over production of whole antigens in cell-based systems due to cost and ease of standardization.

PEP-FOLD_3_ predictions of the secondary structure of FVIII_2155-2180_ showed an α-helix encompassing P2143 to Y2148, corresponding to P3-P8 of the core sequence fitting into the MHC-II groove. P4 and P6 anchor amino acids are part of the helix, but since FVIII_2155-2180_ requires processing before presentation, the rigidity of the helix is not expected to interfere with binding. Moreover, the analysis of the secondary structure guided the choice of coupling Sia in a non-structured end-terminal of the peptide, so that binding of Sia to Siglecs would not be impeded by the rigidity of the aminoacidic scaffold.

Although Pletinckx *et al.* demonstrated that peptide immunotherapy reduced inhibitors in HA mouse models, the development of these antibodies was not impeded (Pletinckx et al., 2022). To enhance efficacy, we proposed to couple the peptides to sialic acids to target Siglecs. It is well established that Siglecs engagement promotes immune tolerance in various contexts, such as controlling excessive inflammation during sepsis (Spence et al., 2015). In HA, targeting Siglec-2 on B cells with sialylated nanoparticles containing FVIII achieved complete tolerance (Macauley et al., 2013). Given that DCs are the main antigen-presenting cells and that the development of inhibitors requires APCs’ licensing, we designed a sialylated saccharide to target Siglec-9, highly expressed on DCs (Figure 3D). While we selected the α2.3-sialylated tetrasaccharide based on MD simulations to Siglec-9 (Figures 2A–C), we also observed binding to Siglec-3 (Figures 3A–C). This is not unexpected, as Siglecs are known for binding promiscuity, and the specific ligands are not yet fully elucidated for all the lectins. Interestingly, targeting Siglec-3 was explored for Alzheimer’s disease, where its suppression of microglial activity was found to be detrimental (Griciuc et al., 2013). However, in the present study Siglec-3 engagement could be advantageous, since it is an inhibitory Siglec and its pattern of expression is mainly restricted to APCs. Despite plate-bound siaFVIII_2155-2180_ was shown to bind Siglec-3 and Siglec-9 (Figures 3A–C), no differences in peptide binding/uptake were observed, regardless of sialylation, on moDCs (Figure 3D; Supplementary Figure S2B-C). While Siglec-1 and Siglec-2 are described to be endocytic receptors (Delputte et al., 2011; O'Reilly et al., 2011), to our knowledge there are no studies linking Siglec-3 and Siglec-9 to improved internalization, their main role being immunosuppression via ITIMs. It is therefore expected that α2.3-sialylation would not improve endocytosis of FVIII-peptides by moDCs. Given the phagocytic function of moDCs, a non-specific uptake of the unsialylated peptide is also expected. However, our detection system with TAMRA as fluorochrome may have lacked sensitivity, and results should be confirmed by a different tracking system.

Interestingly, when we measured Siglecs activation, we could only detect phosphorylated Siglec-9 (Figures 4A,B) and not Siglec-3 (Supplementary Figure S1A-B). This confirms that Siglec-9 will likely be the main target of siaFVIII_2155-2180_, while binding to Siglec-3 might not be strong enough to also induce signalling. Low Siglec-9 phosphorylation (pSiglec-9) was detected in samples treated with FVIII_2155-2180_. Recent research also reported pSiglec-9 in control-treated samples (Rodriguez et al., 2021; Lübbers et al., 2021). These and our data suggest that pSiglec-9 observed with FVIII_2155-2180_ is not induced by the treatment with the peptide but represents a physiological condition of unstimulated monocytes.

Lectins bind glycans with low affinity, with avidity enhancing the interaction (Dam and Brewer, 2010). A multivalent presentation of the ligand is generally advantageous (Gonzalez-Gil and Schnaar, 2021). Since a single sialylated moiety on an aminoacidic sequence may not achieve optimal multivalency, we coated the peptides on a plate for binding and monocyte-stimulation assays, to enhance the sensitivity required for assessing Siglec-triggering *in vitro*.

In the present study, we demonstrated that IL-10 secretion from moDCs can be induced by triggering Siglecs with siaFVIII_2155-2180_ (Figure 4G). While previous research from our group showed that α2.3-sialylated dendrimers can achieve the same effect (Lübbers et al., 2021), the key advantage of our approach is its antigen specificity. By conjugating Sia to an immunodominant-FVIII-derived peptide, we ensured that the tolerogenic signal was directly linked to the antigen towards which tolerance is to be induced. However, further research is needed to confirm this hypothesis. We also demonstrated that IL-10 secretion is dependent on Siglec-3 and Siglec-9, as confirmed by knock-out experiments (Figures 4H–J). We detected low IL-10 secretion in Siglec-3 and double KOs, suggesting that Siglec-3 might play an essential function in moDC biology which cannot be functionally tested with a knockout. We did not observe any differences in cell viability between the nucleofected cells, and we could detect most of the cytokines tested due to the high sensitivity provided by the bead array. Even though IL-10 was the only cytokine significantly altered in the mock moDCs by siaFVIII_2155-2180_ treatment, other cytokines were upregulated that are considered to be pro-inflammatory (IP-10, IL-4, TNF-α, and IFN-γ). However, the maturation and tolerogenic markers were not affected regardless of peptide-treatment or Siglec-expression (Supplementary Figure S1E-H), which is an observation in line with previous research on immune-modulation induced by sialylated glycans (Lübbers et al., 2021). Overall, despite the presence of pro-inflammatory cytokines, the activation status of moDCs was not altered by siaFVIII_2155-2180_, and we measured significant induction of IL-10 secretion. IL-10 is a key cytokine in immune homeostasis, and dysregulations of its pathway have been linked to a number of autoimmune and inflammatory diseases, such as IBD, RA and SLE (Wang et al., 2019). The development of inhibitors is an immune-related complication with an unclear cause, but it shares mechanisms with tolerance breakdown tolerance observed in autoimmunity (Varthaman and Lacroix-Desmazes, 2019). Therapies aiming at restoring IL-10 levels are already widely being investigated for autoimmune diseases (Wang et al., 2019). Moreover, it was shown that IL-10 is a key cytokine to restore tolerance to FVIII in hemophilic mice (Oliveira et al., 2013). Concluding, we suggest that promoting IL-10 secretion from moDCs via Siglec-3 and Siglec-9 with sialylated FVIII-derived peptides as shown in this study could also could help restore a tolerogenic milieu in HA patients that developed neutralizing antibodies to FVIII.

## Data Availability

The original contributions presented in the study are included in the article/Supplementary Material, further inquiries can be directed to the corresponding author.
