# Supplementary material for: Rational design of FVIII sialylated peptides to target Siglec-3 and Siglec-9 and improve peptide formulations for reverse vaccines
Source: Front Bioeng Biotechnol. 2025 Apr 10;13:1558627. doi: 10.3389/fbioe.2025.1558627 (PMC12018916; doi:10.3389/fbioe.2025.1558627)
Supplement: Supplementary file 1 [file DataSheet1.docx]

Supplementary Material

# Supplementary method. Peptide synthesis and conjugation to the sialylated glycan and TAMRA

The functionalized glycan was synthesized by first dissolving Neu5Acα2-3Galβ1-4GlcNAcβ1-3Galβ1-4Glc (LSTd, 50 mg, 49 µmol, 1.0 equiv.) in a mixture of DMSO and acetic acid (3:1, DMSO – AcOH, 2.5 mL, 20 mM) and the solution was warmed to 65°C. Then, 4-(4-N-maleimidophenyl)butyric acid hydrazide (MPBH, 40 mg, 0.11 mmol, 2.3 equiv.) was dissolved in DMSO (0.5 mL, 0.22M) and added dropwise to the reaction mixture, which was then stirred at 65°C for 30 min. Next, picoline borane (67 mg, 0.63 mmol, 13 equiv.) was dissolved in DMSO (0.5 mL, 1.3M) and added dropwise. The reaction mixture was stirred at 65°C for 2 hrs. The solution was cooled down and DCM (8 mL) was added followed by Et_2_O (40 mL) upon which a white precipitate was formed. The suspension was centrifuged and the residue was washed with Et_2_O (40 mL). The pellet was dissolved in water and purified over HPLC (20% B to 60% B) on an InfinityLab Poroshell 120 EC-C18 column (4 µm, 21.2 x 250 mm) to give LSTd-MPBH (18.5 mg, 15 µmol, 30%). Solvent A: 0.1% TFA in H_2_O. Solvent B: 0.1% TFA in MeCN. LSTd was purchased from Elicityl Oligotech and MPBH from Thermo Scientific. For the conjugation of LSTd-MPBH to the FVIII sequence, (4.14 mg, 1.27 µmol, 1.0 equiv.) was dissolved in DMSO (447 µL, 3 mM) and 2,4,6-collidine (3 µL, 23 µmol, 18 equiv.) was added. The solution was then added to LSTd-MPBH (2.0 mg, 1.59 µmol, 1.25 equiv.) and the reaction was shaken for 30 minutes. LC-MS indicated completion, H_2_O (50 µL) was added and the mixture was purified over HPLC (20% B to 50% B) on an Agilent Zorbax C18 column (5 µm, 9.4 x 250 mm) to yield sialylated peptide (4.1 mg, 0.90 µmol, 71%). Solvent A: 0.05% TFA. Solvent B: MeCN. Mass and purity were confirmed by UPLC-MS (1). The peptides were dissolved in sterile water in a concentration of 500 µM. Labelling of peptides with TAMRA on the C-terminal was achieved using standard microwave coupling procedure after final amino acid and prior to cleavage. The peptides were purified on a preparative Ultimate 3000 HPLC system (Thermo Fisher) over a Vydac 218MS1022 C18 25 × 250mm column (Grace Davidson). Confirmation of purity and mass was achieved by the Ultimate 3000 [UHPLC](https://www.sciencedirect.com/topics/immunology-and-microbiology/ultra-high-performance-liquid-chromatography) system (Thermo Fisher) hyphenated with an LTQ Iontrap [ESI](https://www.sciencedirect.com/topics/immunology-and-microbiology/electrospray-ionization) mass spectrometer (Thermo Finnigan) via a RSLC 120 C18 Acclaim 2.2 μm particle 2.1 × 250 mm column and sample ionization in positive mode. Fluorescence intensities of the TAMRA-labelled peptides were checked at the fluorimeter (Synergy HTX) with the following parameter: Excitation 565/10, Emission 590/20, automatic gain.

# Validation of sialylation and binding to Siglecs

One µM of FVIII_2155-2180_ or siaFVIII_2155-2180_ diluted in PBS (Braun) were coated on NUNC maxisorb plates (Roskilde) overnight at room temperature. Ten µg/mL of Neu5Acα3`Lac-Gly-PAA (Merck, 0060-PA) or Neu5Acα6`Lac-C2-PAA (Merck, 0063A-PA) were used as positive controls for α2,3-specific Lectenz and MAL-I and SNA respectively. The wells were then blocked for 30 minutes at 37°C with carbo-free blocking buffer (Vector Laboratories, SP5040). Streptavidin conjugated to horseradish peroxidase (Sigma-Aldrich) allowed spectrophotometric quantification of the binding with 3,3′,5,5′-tetramethylbenzidine (Sigma-Aldrich) at 450 nm on the iMarkTM Microplate Absorbance Reader (Bio-Rad). Similarly, binding to Siglecs was assessed by ELISA with the Siglec-Fc chimeras indicated in Supp. Table 1. As positive controls various glycoforms of PAA polymers (Merck) were used. Plates were blocked with carbo-free blocking buffer (Vector, SP5040) diluted 1:10 with HBSS (Invitrogen) for 30 minutes at room temperature and subsequently incubated with 3.3 μg/ml Siglec-1/-3-/-7/-10-Fc or 2 μg/ml Siglec-15-Fc or 0.2 μg/ml Siglec-9-Fc chimeras in combination with goat anti-human or goat anti-mouse Fc-PO antibody (Jackson, 109-036-098 and 115-036-071). Binding was visualized using TMB followed by measurement at 450 nm.

# Supplementary Table 1

List of Siglec-Fc chimeras used in ELISA.

| **Siglec-Fc** | **Catalogue number** | **Manufacturer** |
| --- | --- | --- |
| Siglec-1 | 5610-SL | R&D systems |
| Siglec-3 | 1137-SL | R&D systems |
| Siglec-7 | 1138-SL | R&D systems |
| Siglec-9 | 1139-SL | R&D systems |
| Siglec-10 | 2130-SL | R&D systems |
| Siglec-15 | 9227-SL-050 | R&D systems |

# Supplementary Table 2

List of antibodies used for flow cytometry stainings of moDCs.

| **Antigen** | **Fluorochrome** | **Clone** | **Manufacturer** |
| --- | --- | --- | --- |
| CD86 | PE | 2331 (FUN-1) | BD Biosciences |
| CD80 | BV605 | L307.4 | BD Biosciences |
| PDL1 | Pe-Cy7 | 29E.2A3 | Biolegend |
| TIM3 | BV510 | F38-2E2 | Biolegend |
| Siglec-9 | AF488 | FAB1139G | R&D Systems |
| Siglec-3 | BV650 | WM53 | Biolegend |

# Supplementary Table 3

List of core peptides of FVIII input into the *in silico* analysis.

| #1 | TVVITLKNMASHPVSLHAVGV |
| --- | --- |
| #2 | AWPKMHTVNGYVNRSLPGLIG |
| #3 | GEVGDTLLIIFKNQASRPYNI |
| #4 | PTKSDPRCLTRYYSSFVNMER |
| #5 | QANRSPLPIAKVSSFPSIRPIYLT |
| #6 | EVEDNIMVTFRNQASRPYSFY |
| #7 | LHAGMSTLFLVYSNKCQTPLG |
| #8 | NPPIIARYIRLHPTHYSIRST |

# Supplementary Figures

**
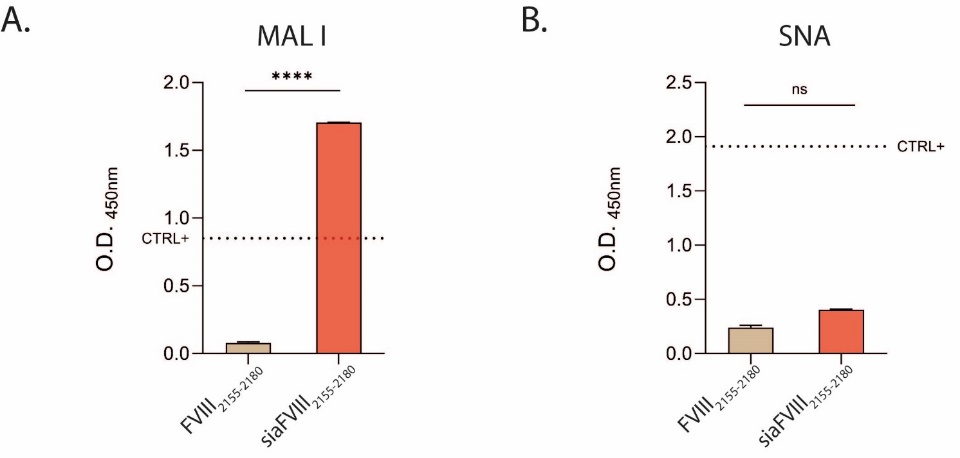
**

**Supplementary Figure 1.** MAL I and SNA binding to siaFVIII_2155-2180_. The dotted line marks the mean O.D. value of α2.3 PAA (A) or α2.6 PAA (B) used as a positive controls. Representative of 3 independent experiments performed in duplicate. Statistics: unpaired t test.


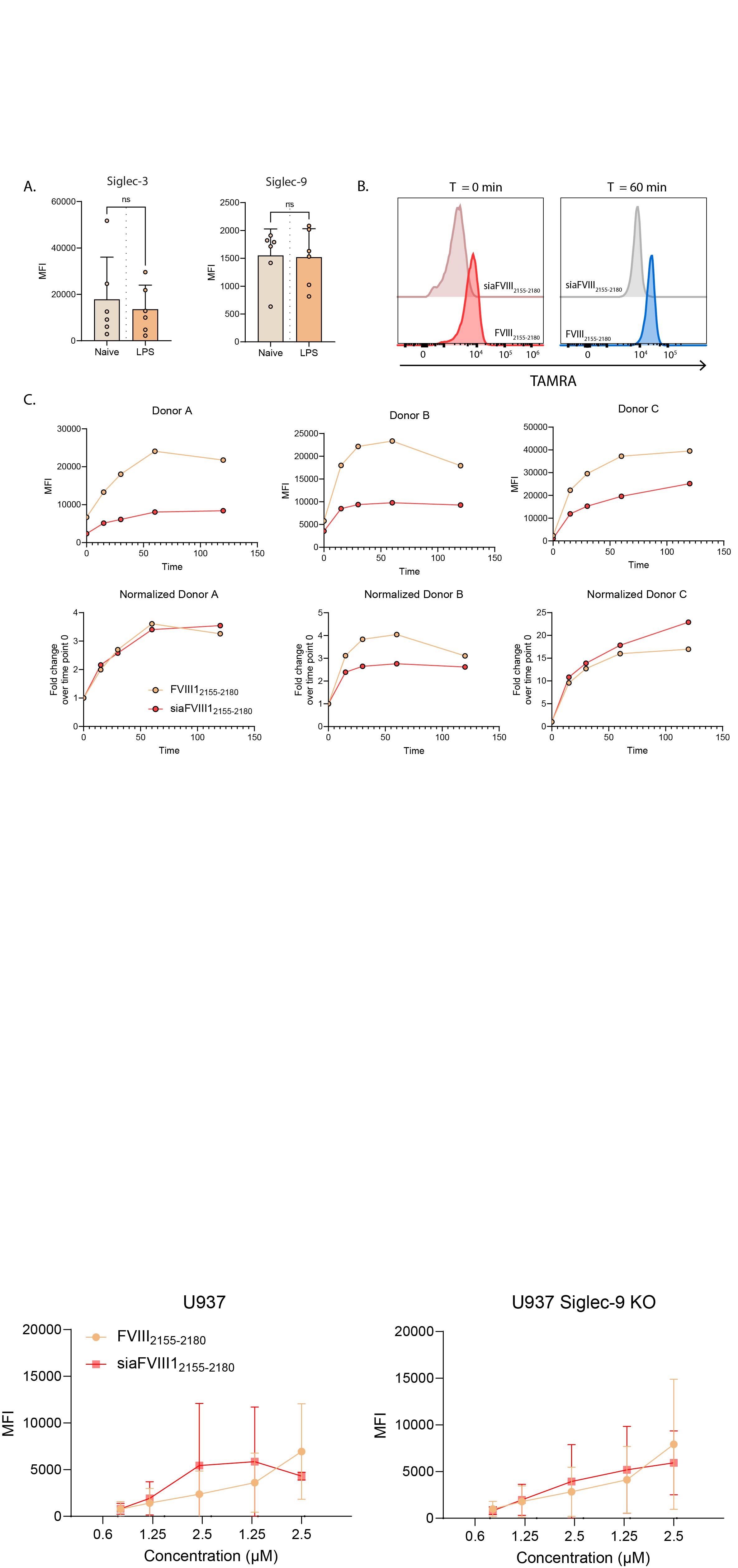


**Supplementary Figure 2.** siaFVIII_2155-2180_ efficiently binds/internalizes in moDCs. A. Median fluorescence intensities (MFI) of Siglec-3 BV650 and Siglec-9 AF488 measured by flow cytometry in unstimulated (naïve) and LPS-treated moDCs. Data are presented as mean ± SD. Each dot represents one donor. N = 6. Statistics: paired t test, ns = not significant. B. moDCs were incubated with 10µM of TAMRA-(sia)FVIII_2155-2180_ and fluorescence was measured by flow cytometry. Representative histograms of one donor showing TAMRA-FVIII_2155-2180_ of TAMRA-siaFVIII_2155-2180_  fluorescence in moDCs at time point 0 (left) and after 60 mins of incubation at 37°C. C. Individual plots are shown for 3 representative donors, as raw MFIs (top) and normalized over time point 0 (bottom).


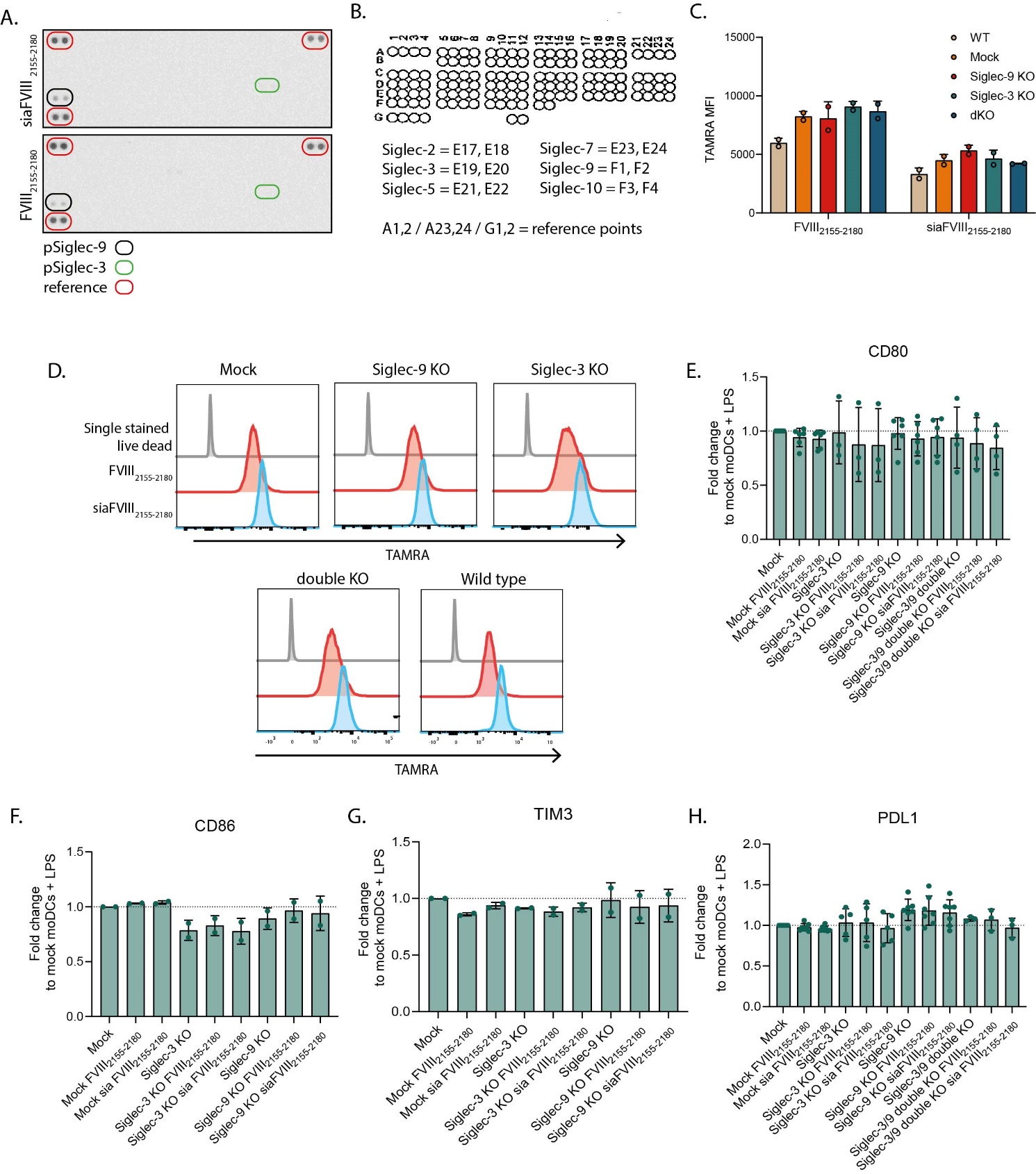


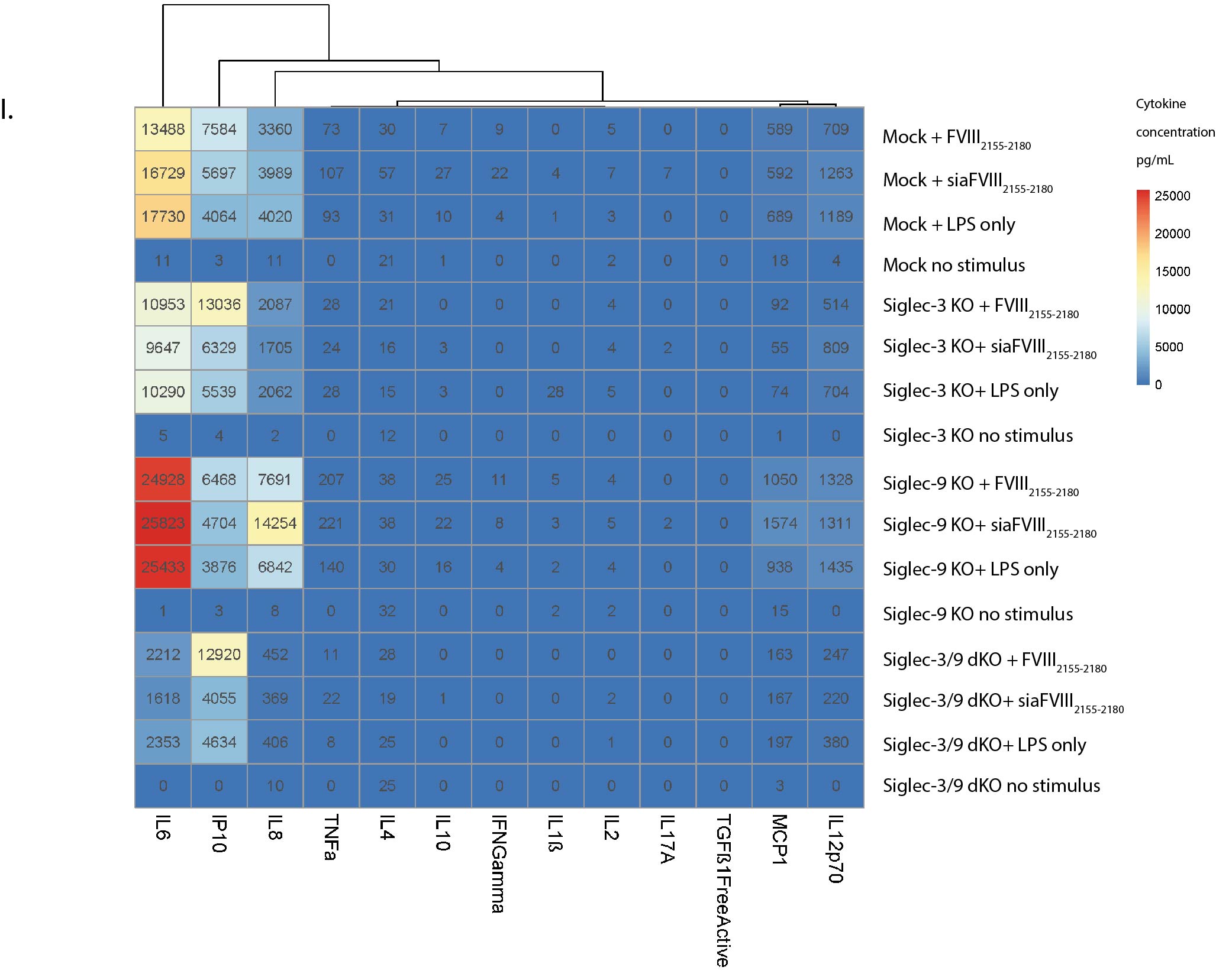


**Supplementary Figure 3.** A. Whole membrane from the Human Phospho-Immunoreceptor Array Kit (R&D systems). B. Reference for the spots on the membrane for Siglecs, as reported in the manufacturer‘s manual. C. Flow cytometry analysis of the median fluoresce intensity of TAMRA in wild type (WT), mock, Siglec-9 KO, Siglec-3 KO moDCs and Siglec-3/9 double KO (dKO) after 30 mins incubation on ice. Each dot represents one donor. D. Flow cytometry analysis of different moDC cell lines incubated with TAMRA-FVIII_2155-2180_ or TAMRA-siaFVIII_2155-2180_ for 30 mins on ice. Data from one donor are shown as histograms of TAMRA fluorescence. E-H. Expression of maturation and tolerogenic markers measured by flow cytometry and plotted as fold change to LPS-treated mock moDCs. Each dot represents one donor. N = 2 – 7 donors. I. Cytokines' concentrations in the supernatant of mock, Siglec-9 and Siglec-3/9 double KO moDCs stimulated with siaFVIII_2155-2180_ or FVIII_2155-2180_ in the presence of LPS. No stimulus indicates the condition where LPS was not added. Each number is the mean of two technical duplicates per donor. For mock and Siglec-9 single KO, N donors = 8, for Siglec-3 single KO, N donors = 4, for Siglec-3/9 double KO, N of donors = 3.
